# Marine resource congestion in China: Identifying, measuring, and assessing its impact on sustainable development of the marine economy

**DOI:** 10.1371/journal.pone.0227211

**Published:** 2020-01-24

**Authors:** Qiang Cao, Caizhi Sun, Liangshi Zhao, Weiwei Cao, Xiaolu Yan

**Affiliations:** 1 Center for Studies of Marine Economy and Sustainable Development, Liaoning Normal University, Dalian, Liaoning, China; 2 College of Urban and Environment, Liaoning Normal University, Dalian, Liaoning, China; Center for International Climate and Environmental Research - Oslo (CICERO), NORWAY

## Abstract

Research on the sustainable development of the marine economy has conventionally revolved around the relationship between efficiency and development. However, most studies have neglected examining how excessive marine resource inputs under certain conditions may lead to resource congestion that restricts output efficiency and sustainable development. To fill this research gap, we optimized an index system to evaluate the input level of marine resources. Using the data of 11 coastal provinces and cities in China from 2000 to 2016, we calculated the congestion of marine resources and analyzed its spatiotemporal evolution and primary influencing factors. Finally, we separated the inefficiency driven by congestion from pure technical inefficiency. The results showed the following: (1) Grave, long-term marine resource congestion does exist in China, and it has evolved from fast to slow, strong to weak, and agglomeration to dispersion; (2) Congestion in the coastal areas has gradually weakened from north to south, and the center of gravity has experienced a shift from the center of China toward the north; (3) Marine resource congestion is mainly affected by the input of resource and capital, resource endowment, and industrial structure; (4) Factors leading to inefficiencies include resource congestion and long-term pure technical inefficiency. By combining congestion and efficiency, we produce values for studying inefficiency and the sustainable development of the marine economy, with the benefit of providing targeted strategies.

## Introduction

Since 2011, the added values of China’s major national marine industries has reached US $2,849.73×10^8^, surpassing that of the United States by US $2,779.02×10^8^, making China the world's largest marine economy [1]. However, there is a massive input of marine resources and damage to the marine ecological environment in China, which in turn has had a negative impact on the economic output efficiency of marine resources [2–4]. At present, although the Chinese government and society have realized the importance of resources and the ecological environment and put forward a series of strict policies for their protection, the government promoted the strategy of “building a maritime power” in The 18th National Congress of the Communist Party of China in 2012 [5, 6]. As the world's largest marine economy, China is expected to exploit and utilize more marine resources, profoundly affecting the world's marine economy, marine resources, and ecological environment [7, 8].

There is a complex relationship and interaction between the input of marine resources and the development of the marine economy [9, 10]. With the development of China's marine economy, the role of marine resources in promoting the growth of the marine economy has been weakened, and the factors supporting the development of China's marine economy have undergone profound changes. From the perspective of the nature of the marine economic system, the main contradiction between the input of marine resources and the marine economy is the efficiency of the transformation of marine resources by marine economic systems. In recent years, scholars have studied the relationship between the input of marine resources and marine economic efficiency [11]. These studies mainly focused on the impact of marine resources on marine economic efficiency, the impact of marine economic efficiency on the utilization of marine resources, and the interplay of marine economic efficiency and marine resources.

In terms of the impact of marine resources on marine economic efficiency, Stock [12] studied the response of marine productivity to climate change with two models, and found that global warming will result in a reduction of marine resources. Zhang [13] calculated the abundance degree of marine resources in Liaoning's coastal areas, and found that the effect of marine resources on the efficiency of the marine economy changed over time. Zhang’s study also showed that marine resources play a foundational role in regional economic development.

In terms of the impact of marine economic efficiency on the utilization of marine resources, Cullinane [14] and Clark [15] explored the impact of the economy on marine space resources, and found that the efficiency of ports is positively affected by marine economy, and greater private-sector participation and with transshipment as opposed to gateway ports. Sun [16] and Gai [17] applied Data Envelopment Analysis (DEA) to study the coupling and coordination degree between input of marine resources and marine economic efficiency from the perspective of environmental regulation, and found that the time series of China' s marine composite system's carrying capacity shows an increasing trend. These studies also found that previous research had overestimated ecological efficiency.

In terms of the interplay of marine economic efficiency and marine resources, Hilborn [18] found that large subsidies to the world's fisheries encouraged overfishing and provides society with a small fraction of the potential economic benefits. Based on Romer’s “drag effect” hypothesis and neo-classical theory of economic growth, Wang [19] developed a model of marine resources consumption drag on the growth of marine economy in China, and found that the restraint of marine resources with marine economic growth is high. Additionally, the growth rates of labor and marine resource consumption; the output elasticity of marine resources; and capital elasticity are proportional to marine resources consumption drag.

In summary, while these studies have usefully examined the interrelationships between marine economic systems and marine resources from various perspectives, they often assume that the increasing input of marine resources will not reduce the output of the marine economy, or that reducing input of marine resources will not increase the output [20–22]. One limitation of this assumption is that it excludes the possibility of further increases in the input of marine resources leading to a decline in economic output. The "congestion effect" often occurs in realistic social and economic activities [23]. In other words, when the input of other production factors is fixed, that of certain variable production factors is excessive, and the marginal revenue becomes negative, the output will decline. This theory of “congestion effect” is primarily based on the law of diminishing marginal returns of production factors, reflecting the imbalance of the proportion of production elements. The related research shows that the development of China's marine economy is based on a large amount of marine resource input.

To address the knowledge gap regarding the relationship between marine resource congestion and efficiency in China, our objective was to identify, measure and assess the impact of marine resource congestion on the sustainable development of the marine economy. Specifically, we aimed to answer the following research questions: 1) Is there any congestion in China's marine resources? 2) What are the characteristics of congestion? 3) What factors affect congestion? 4) How does marine resource congestion influence the efficiency of the marine economy? The rest of this paper is organized as follows. In Section 2, we introduce the characteristics of the study area, the source of the data, and the use of the classical model proposed by Färe (1985) to measure the congestion of marine resources. Based on the model, we introduce a decomposition model to test the inefficiency of marine resources driven by marine resource congestion and pure technical inefficiency. At the same time, we optimized an index system for evaluating the input of marine resources and explaining how the data is processed. In Section 3, we analyze the spatiotemporal characteristics and influencing factors of the measured congestion results and the impact of marine resource congestion on the inefficiency of the marine economy. Section 4 discusses the theoretical contributions and policy implications.

## Materials and methods

### Study area and data sources

**Fig 1:**
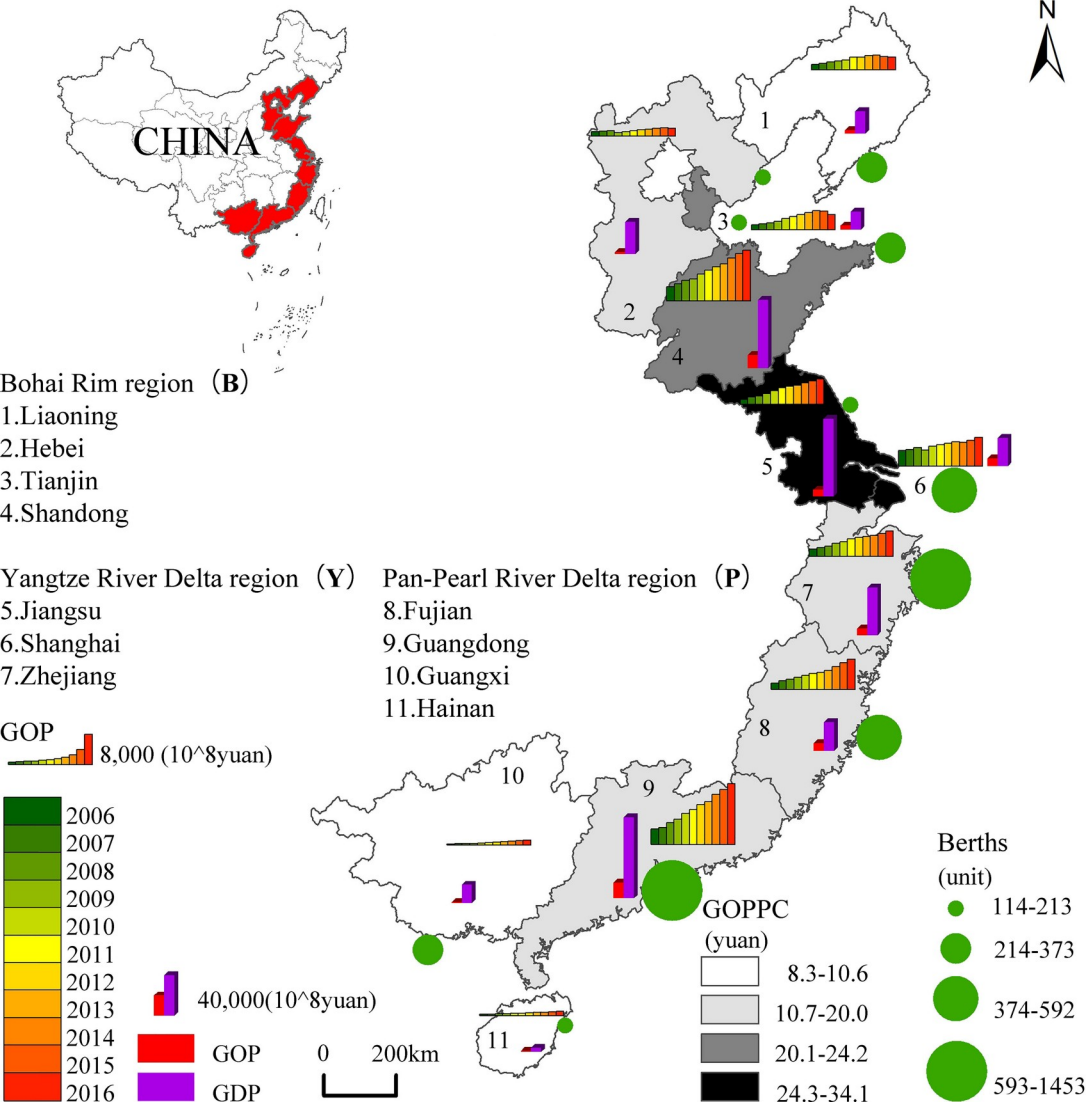
Development level of marine economy of China’s 11 coastal provinces and cities.

In order to determine whether there is marine resource congestion, how congestion leads to the inefficiency of the marine economy, and the spatiotemporal evolution characteristics, China's 11 coastal provinces and cities, except Hong Kong, Macao, and Taiwan, were selected for this research (Fig 1). This study focuses on the 2000–2016 time period. These provinces and cities are frequently divided from the north to the south into the Bohai Rim region, the Yangtze River Delta region, and the Pan-Pearl River Delta region. In 2016, these three regions accounted for 32,000 km total length of coastline, 13.4% of the country’s land mass, a total population of 137.46 million, and GDP of 744,127.2 million yuan (57.12% of total national GDP). Gross ocean product (GOP) was 69,693.7 million yuan, marine fisheries accounted for 4615.4 billion yuan, and marine oil and gas accounted for 868.8 billion yuan. One can find striking spatiotemporal differences, which include GOP, the ratio of GOP to GDP, per capita GOP (PCGOP), and berths for productive use above designed size (Berths).

A panel dataset of 11 regions from 2000 to 2016 is collected for analysis. Marketization index data is collected from the Marketization Index of China’s Provinces: Neri Report 2016. The rest of the data is collected from the China Marine Statistical Yearbook (State Oceanic Administration of China, 2001–2017).

### Methods

DEA is essentially an efficiency evaluation model based on an input-output system. It has been widely used in the study of marine economic efficiency. From the perspective of input-output indicators, the input indicators consider the use of a single resource or multiple resources, while output indicators mainly consider whether to use unexpected output [24]. From the perspective of model selection, early models such as DEA-CCR and DEA-BCC have been gradually developed into correction models with higher accuracy, such as Bootstrap-DEA, Network DEA and Slack-Based Measure [25].

The concept of congestion originated from the area of transportation. It originally refers to the phenomenon of road congestion caused by excessive input of transportation, resulting in the decrease of transportation efficiency. In the field of economics, the economist McFadden (1978) was the first to find that the equal production line appeared as a backward curve, i.e., with the increase of an input factor, the output declined [26]. The congestion effect refers to the phenomenon that the total output decreases when the input of some variable factors of production becomes negative whilst the input of other factors of production remains fixed and unchanged. Its theoretical basis is the law of diminishing marginal returns of production factors, which reflects the imbalance of the structure of production factors. Since Färe and Grosskopf (1983) described input congestion in the DEA framework, input congestion has played an important role in DEA research [27]. According to the results of previous research, the low efficiency of China's marine economy may be due to the excessive input of marine resources and capital, and the low level of marine technology, which leads to the imbalance of various factors in the economy [28]. However, to date there have been no studies of the congestion effect caused by the excessive input of marine resources using the DEA model. In this study, we use a model proposed by Färe [29] to study the occurrence of marine resource congestion. We believe that this method has significant advantages for our study [30]. Firstly, it follows the law of diminishing marginal returns and has a good theoretical basis. Secondly, Farrell's input measure is consistent with the marine resource efficiency evaluation model [31]. Thirdly, it can be conveniently extended to the inefficient decomposition of marine resources related to marine resource congestion.

#### Marine resource congestion measure

Suppose there are *n* decision making units (DMUs), in which each DMU employs non-marine resource input (*X*) and input of marine resource (*M*) to produce output (*Y*), so the production technology can be defined as follows:

T={(X,M,Y):(X,M)canproduce(Y)} (1)

The idea of using the FGL method to test marine resource input is to treat marine resource input strongly and weakly from the perspective of radial model input orientation, and to judge congestion by comparing the differences of efficiency values under different disposal assumptions. First, Model (2) is used to calculate the efficiency value of marine resources under strong disposal input.

$$\begin{array}{l} \min\theta \\ \;s.t.\sum_{j=1}^{n}\lambda_{j}X_{j}\leq \theta X_{0}\\ \;\sum_{j=1}^{n}\lambda_{j}E_{j}\leq \theta X_{0}\\ \;\sum_{j=1}^{n}\lambda_{j}Y_{j}\geq Y_{0}\\ \;\lambda_{j}\geq 0,j=1,2,\cdots ,n \end{array}$$ (2)

Secondly, Model (3) is used to calculate the input efficiency of marine resources under weak disposal.

$$\begin{array}{l} \min\beta \\ \;s.t.\sum_{j=1}^{n}\lambda_{j}X_{j}\leq \beta X_{0}\\ \;\sum_{j=1}^{n}\lambda_{j}E_{j}=\beta X_{0}\\ \;\sum_{j=1}^{n}\lambda_{j}Y_{j}\geq Y_{0}\\ \;\lambda_{j}\geq 0,j=1,2,\cdots ,n \end{array}$$ (3)

The difference between Model (2) and Model (3) is that the resource input of Model (2) is equal sign, and θ* and β* are the optimal solutions of Model (2) and Model (3) respectively. The congestion of DMU can be judged and evaluated by comparing the size of sum. If θ* = β*, there is no congestion; If θ*>β*, then congestion exists. In addition, the degree of congestion can also be expressed by *CE*.

$$CE=1-(\theta^{*}/\beta^{*})$$ (4)

*CE* is between 0 and 1, with a higher *CE* equating to a more serious marine resource congestion effect.

### Marine resource inefficiency decomposition with congestion

In order to further reveal the causes of marine resource inefficiency, we use the decomposition method proposed by Hu and Wang [32] to separate the marine resource inefficiency caused by marine resource input congestion from the global marine resource inefficiency. To achieve this goal, the global marine resource inefficiency is calculated first, which consists of radial inefficiency and slack inefficiency. In addition, according to the decomposition method of Hu and Wang, if the resource input of a DMU is redundant, its improvement can be divided into improvement and relaxation improvement. Among them, radial adjustment (1-θ*), *E*_*0*_ is the optimal solution of Model (2). And the slack adjustment part needs to calculate the improved radial relaxation of marine resource, which can be obtained by solving Model (5):

$$\begin{array}{l} {\mathrm{maxs}}_{e}\\ \;s.t.\sum_{j=1}^{n}\lambda_{j}X_{j}\leq \theta^{*}X_{0}\\ \;\sum_{j=1}^{n}\lambda_{j}E_{j}+s_{e}=\theta^{*}X_{0}\\ \;\sum_{j=1}^{n}\lambda_{j}Y_{j}\geq Y_{0}\\ \;\lambda_{j}\geq 0,j=1,2,\cdots ,n \end{array}$$ (5)

Let Se* be the optimal solution of Model (5), then the inefficiency of global marine resources can be expressed by the ratio of the sum of radial adjustment and slack adjustment to the original marine resource input. And we use ϖ* to represent the inefficiency of global marine resources.

$$\omega^{*}=[(1-\theta^{*})E_{0}+s_{e}{}^{*}]/E_{0}=(1-\theta^{*})+s_{e}{}^{*}/E_{0}$$ (6)

The inefficiency caused by the congestion of marine resources is expressed as:

$$c^{*}=[\beta^{*}E_{0}-(\theta^{*}E_{0}-s_{e}{}^{*})]/E_{0}=\beta^{*}-\theta^{*}+(s_{e}{}^{*}/E_{0})$$ (7)

The pure technical inefficiency can be expressed as:

$$\varphi^{*}=(E_{0}-\beta^{*}E_{0})/E_{0}=1-\beta^{*}$$ (8)

Model (7) and model (8) divided global marine resource inefficiency into two parts, and the decomposition process is shown as follows:

$$\omega^{*}=c^{*}+\varphi^{*}$$ (9)

### Indicators

#### Selection of indicators

If we want to explore whether marine resource congestion exists in the marine economic system, we should first solve the problem of how to effectively evaluate the input of marine resources. Marine resources refer to the material and energy in the ocean and the ocean space related to the exploitation and utilization of the ocean, which can produce economic value under the condition of modern technology [33]. According to natural attributes, marine resources are often divided into marine biological, marine mineral, marine space, and marine tourism resources. China has a wide variety of marine resources with distinct regional characteristics and different levels of development [34]. The economic and ecological values of various marine resources vary greatly, so it is difficult to measure the overall input of marine resources with a single index [33].

China's marine economy is mainly composed of the following industries: coastal tourism; marine communications and transportation; marine fisheries; offshore oil and natural gas; and sea salt, which are also the main industries consuming marine resources. We drew on Wang's[19] study to select indicators from marine biological, marine mineral, marine space and marine tourism resources, as shown in Table 1. The process of index selection in this study uses comprehensive indexes which have overall representational significance for marine resources, to reflect the comprehensive level of China's marine resources development directly, concisely, and comprehensively. Of these, marine nature reserves are the areas of sea, shorelines, and islands delimited for certain marine protections. These are areas of high natural beauty and are an important part of China's marine tourism resources.

**Table 1:** Index system for the input of marine resources.

Criterion	Sub-criterion	Indicator	Type of weights		
			AHP	Entropy	Integrated
**Marine resources**	Marine biological resources	Marine catches production	0.3417	0.0357	0.10081
		Mariculture production	0.0683	0.0500	0.0500
	Marine mineral resources	Offshore crude oil production	0.0996	0.1336	0.1065
		Sea salt production	0.0091	0.1141	0.0769
		Output of marine mining industry	0.0166	0.2081	0.1638
		Output of offshore natural gas	0.0456	0.1460	0.1100
	Marine space resources	Berths for productive use above designed size	0.1590	0.0564	0.0807
		Sea area use management	0.0421	0.0767	0.0942
		Mariculture area	0.1002	0.0551	0.0770
	Marine tourism resources	Number of marine-type reserves	0.0707	0.0632	0.0739
		Number of travel agencies	0.0236	0.0278	0.0264
		Star grade hotels and occupancies	0.0236	0.0331	0.0326

In order to quantitatively calculate the input of marine resources, we normalize each indicator and combine the analytic hierarchy process (AHP) and entropy method to form the integrated weight and use it to calculate the input of marine resources [35]. According to this method, the weights of each type are shown in Table 1.

#### Marine resources data processing

In this study, two non-marine resource inputs, labor and capital, and a single marine resource are used as input variables; GOP is used as the output variable of the marine economic system. The summary statistics of factors are shown in Table 2. Among the factors: (1) Labor refers to those employed in the sea industry in the coastal areas, including the main marine industry personnel and related marine industry personnel. (2) Capital refers to the investment in marine fixed assets. However, since China does not conduct relevant statistics on marine fixed assets, it uses the equal capital output ratio method widely adopted by scholars [36].

$$K=(K_{N}/Y_{N})/Y$$ (10)

Where *K*_*N*_ is the capital stock of 11 coastal provinces and cites, and *Y*_*N*_ is the GDP of 11 coastal provinces and cites, for the year 2000, adjusted at comparable prices. The capital stock is calculated by the perpetual inventory method: *K*_*t*_ *= (1-δ)K*_*t-1*_*+It*, *K*_*t*_ is the capital stock in period *t*, *Kt-1* is the capital stock in period *t-1*, and *δ* is the depreciation rate, which is 9.6% based on the research results [37]. *I*_*t*_ is the new capital stock in period *t*, represented by the total fixed investment. In order to maintain consistency, the data is adjusted according to the fixed asset investment price index based on the year 2000. (3) The input of marine resource was calculated using the above evaluation method and the indicator system in Table 1. (4) GOP is based on statistical data and is adjusted at comparable prices based on the year 2000.

**Table 2:** Summary statistics of input and output factors by area (2000–2016).

Region	Area	Input factors						Output factors	
		Labor (10,000 persons)		Capital (100 million RMB)		The input of marine resource (no unit)		GOP (100 million RMB)	
		Mean	STDev	Mean	STDev	Mean	STDev	Mean	STDev
Bohai Rim region	Liaoning	281.353	299.300	4518.686	3776.688	0.195	0.196	1368.289	1466.556
	Hebei	83.282	88.600	2181.497	2085.218	0.070	0.067	688.569	889.481
	Tianjin	152.612	162.500	3989.687	2738.746	0.102	0.090	1531.203	1335.270
	Shandong	459.147	488.500	9888.895	7474.963	0.312	0.310	3773.653	3779.720
Yangtze River Delta region	Jiangsu	168.906	178.500	4289.203	3022.030	0.098	0.101	1722.025	1494.906
	Shanghai	182.971	194.700	5457.336	6313.056	0.052	0.060	2669.056	3291.815
	Zhejiang	368.047	391.500	5275.143	4518.244	0.242	0.261	2130.692	1961.748
Pan-Pearl River Delta region	Fujian	372.894	396.600	5335.429	3620.672	0.175	0.177	2143.648	1900.500
	Guangdong	725.506	771.600	8319.827	6463.408	0.349	0.365	4551.556	4118.504
	Guangxi	98.800	105.200	937.001	541.319	0.057	0.052	321.343	281.660
	Hainan	115.629	123.100	962.523	663.771	0.070	0.069	324.333	303.718

## Results and discussion

### Marine resource congestion analysis

#### Congestion evaluation

By applying Models (2) through (4), this study calculates the marine resource congestion scores of the 11 coastal provinces and cities of China from 2000 to 2016, and further calculates the congestion averages of the three regions, as shown in Table 3. It can be seen from the table that, in addition to Shanghai, the marine resource congestion in China's coastal areas is widespread, and the congestion in the Bohai Rim region is heaviest. To further explore the spatiotemporal evolution characteristics of marine resource congestion, the following will be analyzed from the perspective of time and space. In order to explore the factors that cause congestion, we conducted a regression analysis of the factors that may result in congestion.

**Table 3:** Marine resource congestion in 11 coastal provinces and cities.

Region	Area	2000	2002	2004	2006	2008	2010	2012	2014	2016	Mean
Bohai Rim region (B)	Liaoning	0.092	0.051	0.019	0.025	0.022	0.028	0.030	0.121	0.164	0.053
	Hebei	0.093	0.055	0.027	0.054	0.024	0.029	0.250	0.317	0.224	0.116
	Tianjin	0.014	0.000	0.010	0.010	0.010	0.130	0.215	0.167	0.201	0.079
	Shandong	0.091	0.058	0.024	0.021	0.019	0.018	0.050	0.204	0.164	0.066
	*Average B*	0.072	0.041	0.020	0.027	0.019	0.051	0.136	0.202	0.188	0.078
Yangtze River Delta region (Y)	Jiangsu	0.100	0.082	0.023	0.016	0.009	0.025	0.130	0.091	0.063	0.061
	Shanghai	0.000	0.000	0.000	0.000	0.012	0.000	0.000	0.000	0.000	0.001
	Zhejiang	0.056	0.000	0.064	0.045	0.040	0.020	0.020	0.007	0.012	0.026
	*Average Y*	0.052	0.027	0.029	0.020	0.021	0.015	0.050	0.033	0.025	0.030
Pan-Pearl River Delta region (P)	Fujian	0.077	0.011	0.011	0.006	0.000	0.000	0.000	0.002	0.077	0.015
	Guangdong	0.013	0.000	0.000	0.006	0.000	0.000	0.002	0.004	0.007	0.003
	Guangxi	0.103	0.067	0.129	0.043	0.031	0.013	0.012	0.014	0.015	0.061
	Hainan	0.109	0.036	0.012	0.347	0.037	0.022	0.011	0.004	0.011	0.047
	*Average P*	0.076	0.028	0.038	0.101	0.017	0.009	0.006	0.006	0.027	0.032

#### Time series analysis

**Fig 2:**
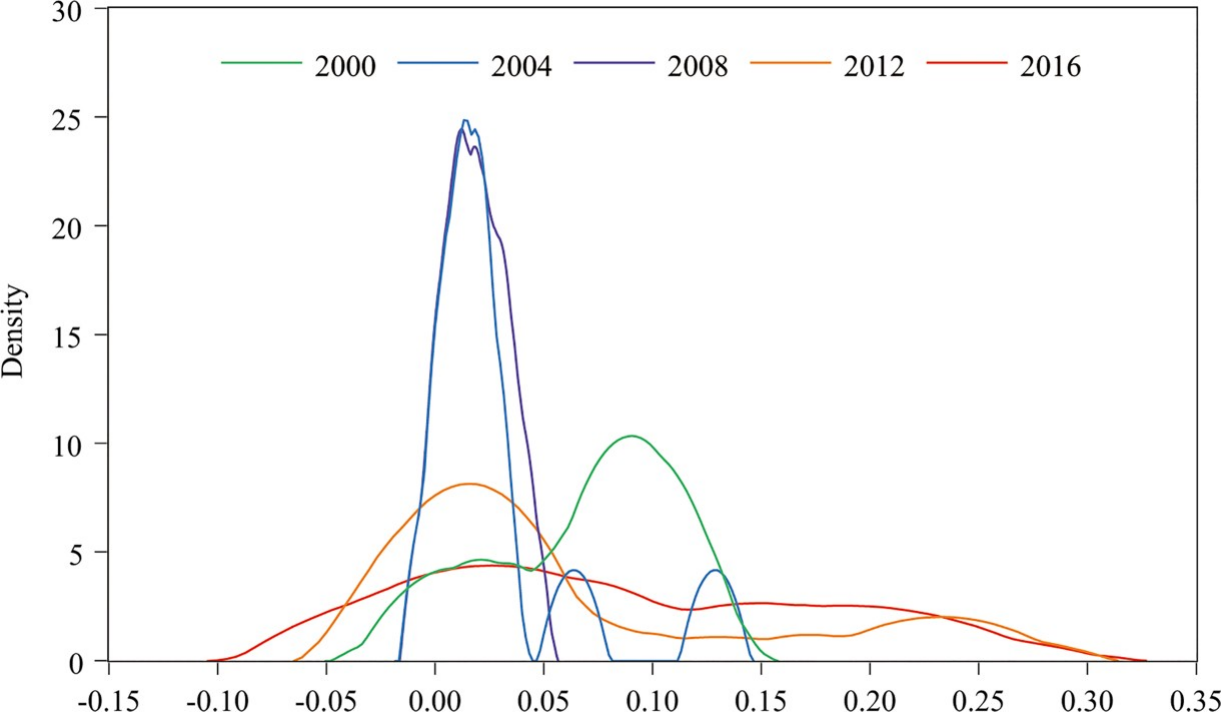
Time series analysis of congestion by kernel density estimation.

To analyze the evolution process of marine resource congestion from the time scale, we selected the congestion results from 2000, 2004, 2008, 2012, and 2016, and incorporated them in the kernel density estimation using Eviews 8.0 (Denver, CO, USA) (Fig 2). Through analysis, we find that it has the following salient features.

First, from the perspective of position, according to the maximum value of the nuclear density to determine the movement of the curve position, it can be seen that the curve generally evolved to the left, between 2000 and 2016, and was most significant in 2000–2004. This indicates that the marine resource congestion in most Chinese coastal provinces and cities decreased significantly from 2000 to 2004, but the change was relatively slow in other stages. In addition, the maximum and minimum values of congestion between 2000 and 2016 showed an overall expansion. The minimum value from 2000 to 2004 increased slightly, and the maximum value remained relatively unchanged. The maximum value from 2004 to 2008 decreased significantly, and the minimum value remained relatively unchanged. The minimum value from 2008 to 2012 decreased significantly, and the maximum value sharply increased. The maximum value for 2012–2016 remained unchanged and the minimum value continued to decrease. The marine resource congestion in China's coastal areas experienced a complex evolution process in 17 years.

Second, from the perspective of shape, there was a trend of change from "chunky type" to "lanky type" and then to "chunky type" in the 17 years, and the change was significant between 2000–2004 and 2008–2012. The change was mostly stable from 2004–2008, with a small change from 2012–2016. These changes show that China's marine resource congestion rapidly converges to low congestion in the early stage, then remains relatively stable before rapidly dispersing, and finally continues to disperse.

Finally, from the perspective of kurtosis, the curve shows an evolution from a narrow peak to a wide peak in 17 years. If the curve is divided according to the maximum kernel density, it can be seen that the area on the left side of the curve was significantly larger than that on the right side in 2000, while the area on the right side of the curve was larger than that on the left side after 2004. This indicates that the marine resource congestion in China generally evolves to be more dispersed. Meanwhile, the marine resource congestion in most provinces was relatively low in the early stage and mainly distributed in relatively high congestion ranges after 2004.

#### Space sequence analysis

**Fig 3:**
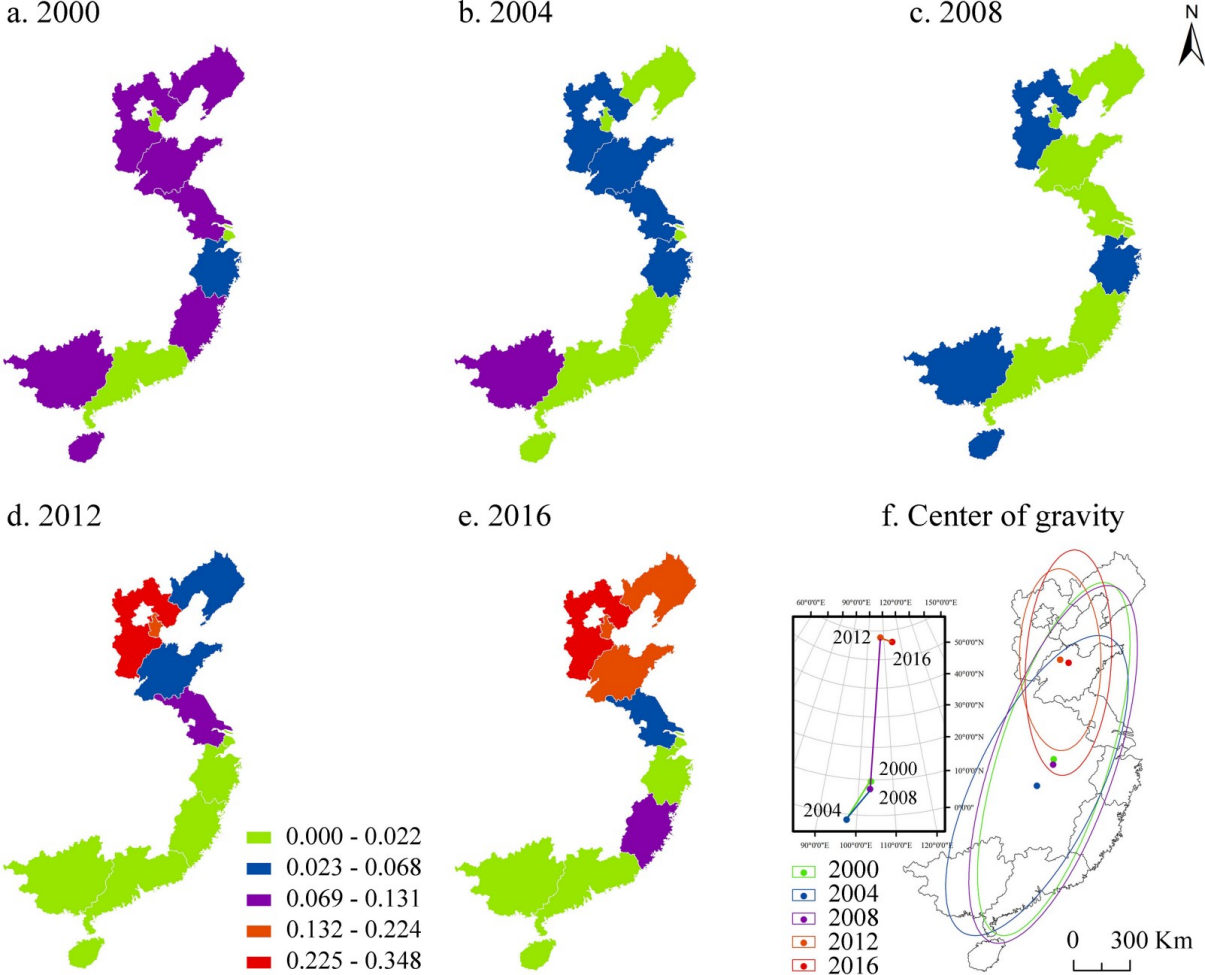
The evolution of the spatial pattern of marine resource congestion.

In order to analyze the spatial evolution characteristics of marine resource congestion, we selected the congestion scores in 2000, 2004, 2008, 2012, and 2016, and used the Jenks natural breaks to divide the congestion score into four categories: extremely low congestion (0.000–0.022), lower congestion (0.023–0.068), moderate congestion (0.069–0.131), higher congestion (0.132–0.224), and extremely high congestion (0.225–0.348). The geographic information system (GIS) spatial analysis technique was used to plot the spatial distribution and center of gravity of the congestion (Fig 3). It can be seen from Fig 3 that the congestion of marine resources was alleviated in some provinces and cities of China and significantly intensified in others, the polarization was significant, and there was a significant spatial agglomeration. In addition, during the 17 years, the center of gravity of the congestion migrated northward, and the standard elliptical area decreased significantly. We find that the center of gravity transfer and standard deviational ellipse have periodic characteristics.

From 2000 to 2004, Liaoning and Hainan fell from moderate congestion to extremely low congestion, while Hebei, Shandong, Jiangsu, and Fujian fell from moderate congestion to lower congestion. These changes caused the center of gravity of congestion in the coastal areas to migrate southward, and the area of standard deviational ellipse was significantly reduced. These illustrate that during this period, the marine resource congestion in the Bohai Rim region and the Yangtze River Delta region was alleviated, causing the congestion of the 11 coastal provinces and cities to shift southward, and the relative size difference of the congestion in the 11 coastal provinces and cities decreased.

From 2004 to 2008, Shandong and Jiangsu fell from lower congestion to extremely low congestion, and from moderate congestion to lower congestion in Guangxi, which caused the center of gravity of congestion to migrate slightly to the north, while the area of standard deviational ellipse increased. This shows that, during this period, marine resource congestion in the coastal areas had become more dispersed, while the congestion in the northern regions had increased slightly.

From 2008 to 2012, Liaoning went from extremely low congestion to lower congestion, Hebei increased from lower congestion to extremely high congestion, and Tianjin increased from extremely low congestion to higher congestion, causing the marine resource congestion in the Bohai Rim region to be significantly intensified. In the same time, Shandong intensified from extremely low congestion to lower congestion, Jiangsu intensified from lower congestion to moderate congestion, and Zhejiang reduced from lower congestion to extremely low congestion, which has intensified marine resource congestion in the Yangtze River Delta region. Meanwhile, the decline from lower congestion to extremely low congestion in Guangxi and Hainan has led to a continued improvement in marine resource congestion in the Pan-Pearl River Delta region. During this period, due to the significant increase in congestion in the Bohai Rim region in the north, congestion in the Yangtze River Delta in the central region increased slightly, and congestion in the Pan-Pearl River Delta region in the southern region rapidly weakened. These factors have caused the congestion of the 11 coastal provinces and cities in China to migrate rapidly from the center to the north, and the area of standard deviational ellipse has been sharply reduced. These changes indicate that during this period, the marine resource congestion in the coastal areas rapidly evolved from south to north and is mainly concentrated in the Bohai Rim region.

From 2012 to 2016, Liaoning and Shandong increased from lower congestion to higher congestion, Jiangsu from higher congestion to lower congestion, and Fujian increased from lower congestion to moderate congestion. These changes have caused the concentration of congestion in the 11 coastal provinces and cities to move slightly downward, and the area of standard deviational ellipse has further narrowed. This illustrates that the marine resource congestion in the Bohai Rim region is further aggravated. The Yangtze River Delta and the Pan-Pearl River Delta region are slightly volatile, but still at a lower level.

### Regression analysis of congestion

There are complex factors influencing the evolution of the spatial pattern of marine resource congestion. Based on the availability of data and the existing research results, this study intends to determine explanatory variables from the following six aspects: (1) per capita input of marine resources involved in the sea (*X*_*1*_), which represents the per capita marine resource input level, as well as the input structure of marine resource and labor resource; (2) per capita marine capital investment (*X*_*2*_), with marine capital investment the main driving force to promote the input of marine resources; (3) per capita coastline length (*X*_*3*_), representing the size of marine resource endowments, which are the foundation of marine resource input and may induce “resource curse,” in various regions. (4) proportion of output value of marine tertiary industry (*X*_*4*_), with the larger the proportion of tertiary industry and more mature the system, the smaller the input of marine resources and the higher the efficiency of the marine economy; (5) marketization index (*X*_*5*_), obtained for 2000–2016 by using the data and measurement methods in the report of Market index by province in China (2016) to represent the level of regional marketization—marketization level is the external environment for the development of the marine economy and the macro condition for the efficiency of marine resources; and (6) the proportion of scientific research personnel per million sea-related employees (*X*_*6*_), used to represent the size of scientific research input in the marine economic system and the science and technology level of the marine economic system. The explained variable is the marine resource congestion calculated in this study. Due to large differences in explanatory variables, we normalized the above data.

The mixed tobit model and the random tobit model were used on the data using Stata15.1. The random tobit model results of random effects LR test results strongly rejected “*H*_*0*_:*ɓ*_*u*_ *= 0*”. To ensure that there is an individual effect, we used the panel tobit regression of random effect for analysis, and the model is as follows:

$$Y=\beta_{0}+\beta_{1}X_{1}+\beta_{2}X_{2}+\beta_{3}X_{3}+\beta_{4}X_{4}+\beta_{5}X_{5}+\beta_{6}X_{6}+\varepsilon$$ (11)

In the formula, *Y* is the degree of marine resource congestion, *X*_*1*_*-X*_*6*_ is the explained variable, and *ɛ* is the random disturbance term. Regression analysis results (Table 4) show that:

**Table 4:** Results of the regression analysis.

C	Coef.	Std.Err	z	p>z
*X*_*1*_	0.432***	0.032	13.37	0.000
*X*_*2*_	0.160***	0.021	7.45	0.000
*X*_*3*_	0.046**	0.018	2.56	0.010
*X*_*4*_	-0.165***	0.023	-7.09	0.000
*X*_*5*_	-0.037	0.022	-1.64	0.101
*X*_*6*_	0.022	0.028	1.39	0.163
*ε*	-0.018	0.014	-1.29	0.197

Note

** and *** respectively represent the significance levels of 5%, and 10%.

(1) Per capita input of marine resources involved in the sea, per capita marine capital input, and per capita coastline length have significant positive effects. The excess input of marine resources is the direct cause of marine resource congestion, the marine capital investment provides capital support for accelerating the input of marine resources, and the marine resource endowment provides natural conditions for the input of marine resources. Therefore, in order to avoid further aggravating the congestion of marine resources, governments at all levels should impose restrictions on the input of marine resources.

(2) The proportion of output value of marine tertiary industry shows a significant negative effect, indicating that the more mature the marine economic system is, the less it relies on resources and the higher the utilization efficiency of marine resources. Therefore, in order to continue the acceleration of the development of the marine economic system, we should pursue the transformation of the marine primary and secondary industries to the tertiary industry.

(3) The marketization index has a negative effect, and the proportion of scientific research personnel per million sea-related employees has a positive effect, but none of them has passed the significance test.

Therefore, the decrease of congestion in these provinces and cities between 2000 and 2016 was due to a gradual improvement of technology driven by growth in the regional economy. In the early stages of this period, there was rapid economic development of coastal regions, promoting a large amount of investment in the marine industry and intensifying the input of marine resources. However, the relatively undeveloped technological level in the early stages led to the imbalance of the input factor structure of marine resources, leading to the high overall level of congestion. In the middle and late stages, with the rapid development of the regional economy, significant improvement of the technological level, and continuous introduction of environmental protection policies, the marine economy has been transformed from resource dependence to technology dependence, which has led to the continuous decrease of congestion. On the other hand, the cluster of spatial congestion is due to the continuous strengthening of concentration of China's coastal economy in the Bohai Rim, Yangtze River Delta, and Pan-Pearl River Delta regions, and the difference of governmental policies in these three regions. The center of gravity of congestion shifted from the center to the north mainly because the transformation of the regional economy, the marine economy, and the technological level was slower in the north than in the central regions. As a result, congestion in the north was relatively increased.

### Marine economic inefficiency analysis

**Fig 4:**
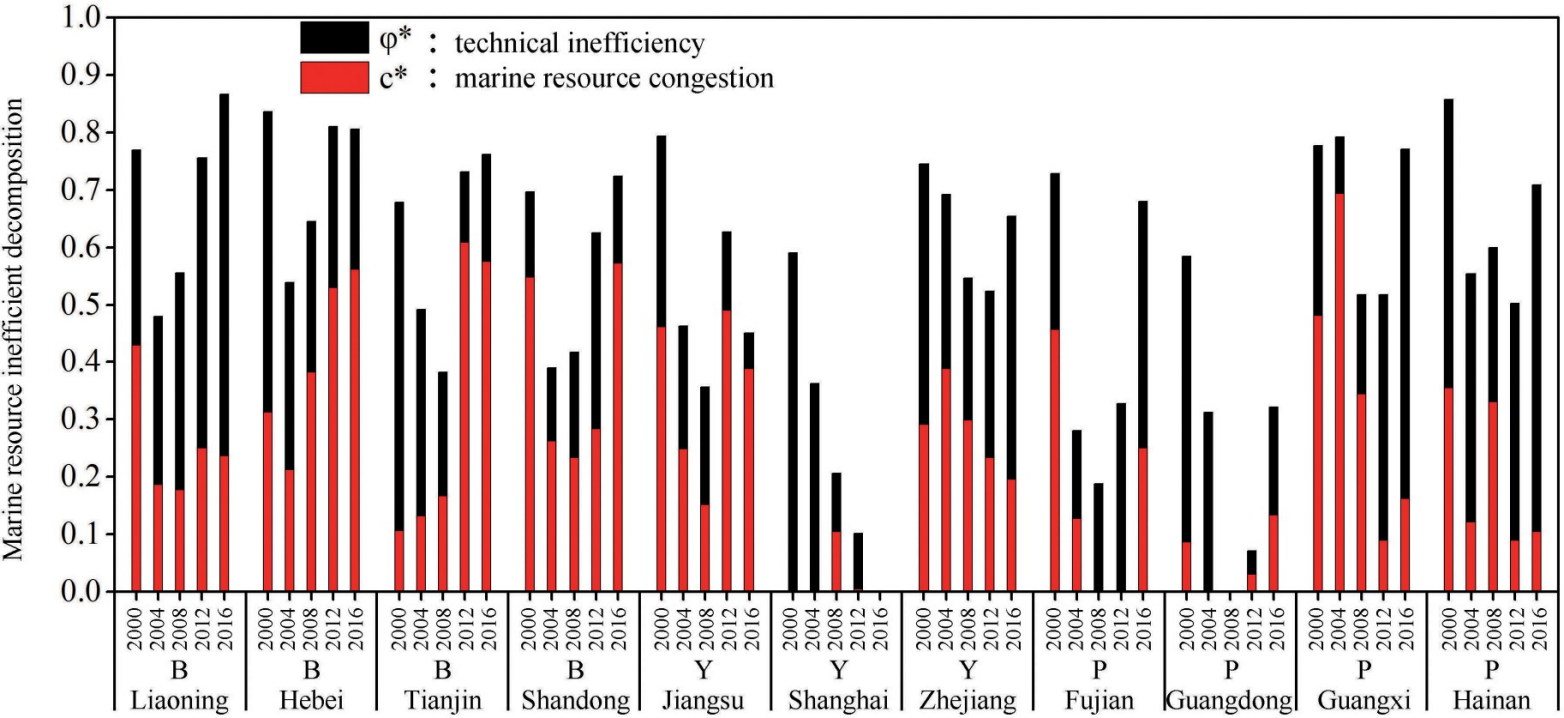
Marine resource inefficiency decomposition for 11 coastal provinces and cities.

Two components of marine resource inefficiency, c*and ϕ*, representing the inefficiency driven by marine resource congestion and pure technical inefficiency, are calculated in accordance with Model (7) and Model (8), respectively. In order to explore the marine resource inefficiency driving mechanism of the three coastal regions, we divided the period from 2000 to 2016 into four stages and calculated the average value (Table 5). Concurrently, in order to intuitively show the driving mechanism and evolution process of the marine resource inefficiency in each region, we selected the data of 2000, 2004, 2008, 2012, and 2016 to draw the histogram (Fig 4). Through the marine resource inefficiency decomposition, we find that the inefficiency of China's marine economy is not only caused by pure technical inefficiency, but also by too much input of marine resources. Further, the leading factors of marine resource inefficiency in different regions and periods also have different state transitions between marine resource congestion and pure technical inefficiency.

**Table 5:** Congestion and inefficiency decomposition in China's three coastal regions.

Area	Year	Congestion	w^*^	c^*^	φ^*^
Bohai Rim region (B)	2000–2003	0.047	0.654	0.291	0.363
	2004–2007	0.023	0.482	0.225	0.257
	2008–2011	0.048	0.612	0.306	0.306
	2012–2016	0.172	0.764	0.493	0.272
Yangtze River Delta region (Y)	2000–2003	0.033	0.603	0.187	0.416
	2004–2007	0.021	0.391	0.175	0.216
	2008–2011	0.023	0.392	0.192	0.200
	2012–2016	0.038	0.396	0.220	0.176
Pan-Pearl River Delta region (P)	2000–2003	0.057	0.630	0.284	0.345
	2004–2007	0.051	0.453	0.285	0.168
	2008–2011	0.012	0.328	0.114	0.213
	2012–2016	0.011	0.489	0.093	0.396

Based on the average value, we can find that marine resource congestion in the Bohai Rim region has intensified, forcing its marine resource inefficiency to deepen. The marine resource inefficiency of the Yangtze River Delta region has increased slightly, and its inefficiency factor has gradually shifted from pure technical inefficiency to marine resource congestion. The marine resource inefficiency of the Pearl River Delta region continues to decline, and its inefficiency has long been caused by pure technical inefficiency.

From 2000 to 2003, the Yangtze River Delta region had the largest congestion of marine resources, and the marine economy in the Bohai Rim region was the most inefficient. During this period, the marine resource inefficiency of the Bohai Rim and the Yangtze River Delta region was mainly caused by pure technical inefficiency, while that of the Pan-Pearl River Delta region was mainly caused by marine resource congestion. From 2004 to 2007, the Pan-Pearl River Delta region had the largest marine resource congestion, and the marine economy in the Bohai Rim region was the most inefficient. In addition, during this period, the marine resource inefficiency of the Bohai Rim and the Yangtze River Delta region was mainly caused by pure technical inefficiency, and that of the Yangtze River Delta region was mainly caused by marine resource congestion.

From 2008 to 2011, the congestion and marine resource inefficiency in the Bohai Rim region was the largest, and the inefficiency was driven by pure technical inefficiency and congestion. The marine resource inefficiency in the Yangtze River Delta and the Pan-Pearl River Delta region was mainly driven by pure technical inefficiency.

From 2013 to 2016, the marine resource congestion and marine resource inefficiency in the Bohai Rim region were significantly higher than in other regions, and its marine resource inefficiency was mainly caused by congestion. The marine resource inefficiency in the Yangtze River Delta region was mainly caused by congestion, while the Pan-Pearl River Delta region was mainly caused by pure technical inefficiency.

According to the marine resource inefficiency of each region (Fig 4), that of Liaoning in the Bohai Rim region is generally on the rise. In 2000, its marine resource inefficiency was mainly caused by congestion, and after 2004, it was mainly caused by pure technical inefficiency. At the same time, the inefficiency caused by congestion remained stable during 2004–2016, while the inefficiency caused by pure technical inefficiency increased significantly. Hebei's marine resource inefficiency showed a downward trend. In 2000, its inefficiency was at its highest level, mainly caused by pure technical inefficiency. With the continuous improvement of marine technology in Hebei, the inefficiency caused by pure technical inefficiency decreased between 2000 and 2016 and increasing resources led to further congestion and significantly restricted the efficiency of the marine economic system. The marine resource inefficiency of Tianjin is generally on the rise. Between 2000 and 2008, the continuous improvement of the technical level made the inefficiency continue to decline, and the increase in the input of marine resource drove the increase in congestion. From 2008 to 2016, the increase in the input of marine resources made the resource congestion exceed the pure technology, and made the overall inefficiency rise rapidly. Shandong's marine resource inefficiency was mainly affected by congestion due to long-term concerns about the development of marine technology.

The marine resource inefficiency of Jiangsu in the Yangtze River Delta region has shown a downward trend. The inefficiency caused by pure technical inefficiency is declining, while the inefficiency caused by congestion shows great fluctuations. Shanghai's marine resource inefficiency shows a steady decline, and its inefficiency has long been caused by pure technical inefficiency. The marine resource inefficiency of Zhejiang shows a downward trend, and the impact of congestion fluctuates. However, the impact of pure technical inefficiency declines in 2012 but intensifies after 2012.

The marine resource inefficiency of Fujian in the Pan-Pearl River Delta region showed a slight decline, and the inefficiency was mainly driven by pure technical inefficiency for several years. Guangdong's marine resource inefficiency shows a significant decline: the inefficiency is mainly driven by pure technical inefficiency, and it fluctuates with time. The marine resource inefficiency of Guangxi is fluctuating, with inefficiency mainly driven by congestion between 2000 and 2008, and by pure technical inefficiency from 2012 to 2016. Hainan's marine resource inefficiency shows a downward trend, and its inefficiency is mainly caused by pure technical inefficiency.

## Conclusions and suggestions

At present, there is a lot of research on the efficiency and development of the marine economic system. Most studies assume that increasing the input of marine resources will not reduce the output of the marine economy or reducing the input of marine resources will not increase the output. However, these studies preclude the possibility that further increases in input of marine resources will reduce the output. We believe that the inefficiency of China's marine economy is directly related to the massive input of marine resources against the backdrop of underdeveloped technology. Based on this, we introduced the "congestion effect" from economics into the study of marine economic efficiency. We used this to explore whether there is marine resource congestion in China's marine economic system, and to find the characteristics of spatiotemporal evolution and the factors affecting congestion, and then to explore the relationship between marine resource congestion and marine resource inefficiency. Through the empirical analysis of the marine economy of 11 coastal provinces and cities in China from 2000 to 2016, the main conclusions and implications derived from the above analysis are provided below.

First, marine resource congestion of 11 coastal provinces and cities in China has long been prevalent. Between 2000 and 2016, the congestion evolved from fast to slow, from strong to weak, from agglomeration to dispersion. Currently, marine resource congestion is still widespread across many provinces and cities, with these provinces and cities continuing to increase their input of resources. The differences between regions in this respect are still significant.

Second, clusters of provinces exhibit similar characteristics and levels of marine resource congestion. These clusters can be divided into the Bohai Rim region, the Yangtze River Delta region, and the Pan-Pearl River Delta region. Additionally, the center of gravity of coastal areas experienced a process of shifting from the center to the north, and the most significant one was from 2008 to 2012. The input of marine resources in the Bohai Rim region continues to increase, so the congestion in the Bohai Rim region is heaviest.

Third, according to the analysis of the affecting factors of marine resource congestion, excessive input of marine resources directly causes congestion. The investment of marine capital further accelerates the development of marine resources. The endowment of marine resources provides a natural foundation for the exploitation and utilization of marine resources. Furthermore, the more mature the marine economic system is, the larger proportion the tertiary industry will be, and the smaller the impact on congestion will be. However, the marketization level provides an environment with less impact on the input of marine resources, and marine science and technology do not play a significant role due to the low conversion rate.

Fourth, China's marine resource inefficiency was severe, but the coastal areas as a whole were slightly relieved during the study period. Among them, the marine resource inefficiency of the Yangtze River Delta region and the Pan-Pearl River Delta region has been alleviated, while the Bohai Rim region has significantly deteriorated. In accordance with the inefficient decomposition, we know that the factors leading to inefficiency in the Bohai Rim region and the Yangtze River Delta region are gradually shifting from pure technical marine resource inefficiency to resource congestion. The inefficiency of the Pan-Pearl River Delta region has long been dominated by pure technical factors, and this continued to increase.

Fifth, the marine resources congestion reflects the structural efficiency of the input factors of the marine economic system. Considering the relationship between resource input and the efficiency of the marine economy, China's marine economy was far behind in the early stage of technology, although its development was improved by a large amount of input in marine resources. Additionally, the resource congestion caused by the massive input of marine resources is an important reason for the low efficiency of the marine economy. In the later stage of the development of the marine economy and related technologies, the inefficiency of the marine economy is caused by the combination of pure technical factors and resource congestion. Our study finds that in the context of technological underdevelopment and imbalanced input factors, too much input of marine resource will hinder efficiency and restrict the sustainable development of the marine economy.

Sixth, the Chinese government should formulate targeted and appropriate development strategies and policies for each of the three coastal regions, in order to alleviate the congestion and improve the efficiency of the marine economic system, to maintain marine economy sustainable development. It is suggested that a more stringent marine resource exploitation and utilization policy should be introduced to address the current situation of severe congestion of marine resources in the Bohai Rim region. In addition, a more open employment environment should be created to reduce the amount of marine resources input from the total amount and structure. It should also limit the construction of enterprises with high resource consumption and high environmental pollution and guide the transformation of marine primary and secondary industries to the tertiary industry. Moreover, we recommend that the Yangtze River Delta region further optimize the marine industry structure on the basis of existing infrastructure and rely on the advantages of local capital, and actively support the emerging strategic industries such as marine biomedicine, marine new energy, and other high-tech and high-capital investment. The Pan-Pearl River Delta region should expand further to open markets for the marine economy, increase the development of marine tourism, and introduce and benefit from the marine science and technology in the Yangtze River Delta and the Bohai Rim regions.
